# A pragmatic cluster randomised controlled trial of a tailored intervention to improve the initial management of suspected encephalitis

**DOI:** 10.1371/journal.pone.0202257

**Published:** 2018-12-06

**Authors:** Ruth Backman, Robbie Foy, Peter J. Diggle, Rachel Kneen, Ava Easton, Sylviane Defres, Fiona McGill, Benedict Daniel Michael, Tom Solomon, on behalf of the ENCEPH UK Programme Steering Committee

**Affiliations:** 1 Department of Clinical Infection, Microbiology and Immunology, Institute of Infection and Global Health, University of Liverpool, Liverpool, United Kingdom; 2 Institute of Applied Health Research, University of Birmingham, Birmingham, United Kingdom; 3 Leeds Institute of Health Sciences, University of Leeds, Leeds, United Kingdom; 4 Lancaster Medical School, Lancaster University, Lancaster, United Kingdom; 5 Department Epidemiology and Population Health, Institute of Infection and Global Health, University of Liverpool, Liverpool, United Kingdom; 6 Department of Neurology, Alder Hey Children’s NHS Foundation Trust, Liverpool, United Kingdom; 7 The Encephalitis Society, Malton, North Yorkshire, United Kingdom; 8 Royal Liverpool and Broadgreen University Hospitals Trust, Liverpool, United Kingdom; 9 NIHR Health Protection Research Unit in Emerging and Zoonotic Infections, University of Liverpool, Liverpool, United Kingdom; 10 The Walton Centre NHS Foundation Trust, Liverpool, United Kingdom; Weill Cornell Medical College in Qatar, QATAR

## Abstract

**Objective:**

To determine whether a tailored multifaceted implementation strategy improves the initial management of patients with suspected encephalitis.

**Design:**

Pragmatic two arm cluster randomised controlled trial.

**Setting:**

Hospitals within the United Kingdom.

**Participants:**

Twenty-four hospitals nested within 12 postgraduate deaneries. Patients were identified retrospectively by searching discharge, microbiology, radiology and pharmacy records and included if they met clinical criteria or had a recorded suspicion of encephalitis.

**Intervention:**

An implementation strategy designed to overcome barriers to change, comprising local action planning, education and training, feedback on performance, a lumbar puncture pack and a range of optional components.

**Outcomes:**

The primary outcome was the proportion of patients with suspected encephalitis undergoing diagnostic lumbar puncture within 12 hours of admission and starting aciclovir treatment within six hours. Secondary outcomes included the proportions of adults and children who had a lumbar puncture, who had appropriate cerebrospinal fluid investigations, and who had appropriate radiological imaging within 24 hours of admission. Data were collected from patient records for 12 months before and 12 months during the intervention period, and analysed blind to allocation.

**Results:**

13 hospitals were randomised to intervention and 11 to control (no intervention), with 266 and 223 patients with suspected encephalitis identified respectively. There was no significant difference in primary outcome between intervention and control hospitals (13.5% and 14.8% respectively, p = 0.619; treatment effect -0.188, 95% confidence interval -0.927 to 0.552), but both had improved compared to pre-intervention (8.5%).

**Conclusion:**

The improvement in both intervention and control arms may reflect overall progress in management of encephalitis through wider awareness and education.

**Trial registration:**

Controlled Trials: ISRCTN06886935.

## Background

There is accumulating evidence that the clinical management of patients with suspected central nervous system (CNS) infections does not meet recommended standards, resulting in lost years and reduced quality of life [1–5]. In the United Kingdom (UK), encephalitis affects between five to nine people per 100,000 every year [6] and is most commonly caused by herpes simplex virus (HSV) type 1 [7–9]. Other causes, including antibody-associated encephalitis, are being recognised increasingly [10]. HSV encephalitis is treated with aciclovir and prompt treatment significantly improves patient outcomes [11–13]. Long-term outcomes from encephalitis are still not understood fully but sequelae include disabilities such as concentration difficulties, behavioural and speech disorders, memory loss and epilepsy [14–16], all of which impact on return to work [15].

HSV encephalitis is relatively rare but patients presenting with clinical features consistent with suspected encephalitis frequently seek medical advice [8]. Encephalitis typically presents with one or more of headache, fever, new-onset seizures, altered consciousness, and behavioural disturbances [17,18]. However, its variable and non-specific features often result in delayed diagnosis and treatment, especially in children who may only present with fever and irritability [5,19]. In addition, delays in performing one of the main diagnostic techniques, a lumbar puncture, may further hamper treatment [20–24]. Previous studies have noted delays between two and 408 hours [2, 11], possibly due to a lack of training, difficulty in finding appropriate equipment, and delays for a computerised tomography (CT) scan [24]. Treatment with aciclovir is also often delayed, with admission to treatment times ranging from 30 minutes to 432 hours after admission [1,2,11] although the reasons behind this are less clear.

Clinical guidelines have been developed in response to these concerns [8,25]. However, dissemination of guidelines alone does not usually bring about significant changes in clinical practice [26–28]. There is a growing evidence base on interventions to promote guideline implementation, but it remains difficult to predict with any confidence which intervention will work best for a given context and targeted behaviour [29]. Ideally, interventions to improve clinical behaviour should be tailored according to identified barriers and needs, preferably focusing on those most amenable to change [30]. Such strategies are often multifaceted, combining different interventions with the intention of targeting a range of barriers, although they are not necessarily more effective than single interventions [31].

The UK Medical Research Council advocates a systematic approach to the development and evaluation of such complex interventions [32,33]. We developed a tailored multifaceted implementation strategy to promote adherence to national guidelines on the initial management of suspected encephalitis [30,34] and evaluated its effectiveness within a cluster randomised trial.

## Methods

### Study design

We conducted a cluster randomised controlled trial with National Health Service (NHS) postgraduate deaneries as the unit of randomisation [35]. Deaneries are responsible for postgraduate medical training.

### Participants

#### Hospitals

This trial took place in the context of a wider research programme *Understanding and Improving the Outcome of Encephalitis* (ENCEPH UK) which comprised several studies. To reduce the likelihood of any unintended co-intervention effects we sought hospitals not directly participating in other ENCEPH UK studies. Sites had to have facilities to perform lumbar punctures and neuroimaging and be willing to be randomised to intervention or control arms. We recruited a range of hospitals providing secondary and tertiary (specialist) adult and paediatric care, to broadly represent national provision and ensure generalisable findings.

We were aware that trainee doctors, one key target intervention group, work and rotate between different hospitals within postgraduate deaneries. We therefore used deaneries as the unit of randomisation as randomising hospitals to intervention and control arms within the same deanery might have risked contamination.

We assessed all 266 acute hospital trusts in England, Wales and Scotland for eligibility. After excluding 47 participating in other ENCEPH UK studies and 10 specialist hospitals not usually providing routine care for suspected encephalitis patients (e.g. orthopaedics) we invited 209 hospitals to participate via senior members of medical staff.

#### Patients

We identified patients with features suggestive of suspected encephalitis using criteria adapted from previous studies [1,9].

- Acute or sub-acute (less than four weeks) alteration in consciousness, cognition, personality or behaviour persisting for more than twenty-four hours. Personality or behaviour change included agitation, psychosis, somnolence, insomnia, catatonia, mood liability, altered sleep pattern and (in children) new-onset enuresis or irritability. Plus any two of:
  - fever (≥38°C) or prodromal illness—acute or sub-acute
  - new-onset seizures; focal neurological signs of acute or sub-acute onset, including focal weakness, oromotor dysfunction, movement disorders (chorea, athetosis, dystonia, hemiballismus, stereotypies, orolingual dyskinesia and tics) Parkinsonism (bradykinesia, tremor, rigidity and postural instability) and amnesia
  - pleocytosis (cerebrospinal fluid [CSF] white cell count of more than four cells per microliter)
  - neuroimaging compatible with encephalitis
  - electroencephalogram (EEG) compatible with encephalitis
- Clinical suspicion of encephalitis noted during the index presentation.
- Clinical suspicion of encephalitis, and the patient died before investigations were completed.

### Intervention

The intervention development and content has been published previously [30]. In brief, using theoretically informed semi-structured interviews based upon the Theoretical Domains Framework [36,37], we explored barriers and enablers to the recommended initial management of suspected encephalitis, specifically performing lumbar punctures and initiating antiviral therapy within 6 hours. This framework has previously been used to understand clinical behaviour across a wide range of healthcare settings [37–45]. We matched behaviour change techniques [34] targeting clinicians to the most salient barriers and enablers and embedded them within an implementation package [30]. The implementation package comprised core interventions, delivered to all hospitals, and optional interventions, which hospitals could use depending on locally available resources and skills. Core interventions included educational and action planning meetings, feedback of pre-intervention audit data and provision of lumbar puncture kits within refillable boxes. Optional interventions included decision support via phone apps and algorithms, an online quiz, prompts and posters, personalised invitation letters to attend educational meetings and a quality improvement cycle pack (materials available via http://www.braininfectionsuk.org/RCTWebsite/). We presented the package at a one-day meeting of senior doctors and nurses from intervention hospitals. These clinical leads generally represented a range of specialties, mainly paediatrics, general medicine, neurology, infectious disease and microbiology, with varying levels of interest and expertise in brain infections. We emphasised their roles in directly delivering the various intervention components locally and recommended that they each convene an action planning meeting on return to their hospitals. All intervention sites received on-going support and materials by a researcher (RB) in addition to the core interventions. This was a pragmatic trial and all intervention sites could choose to what extent they engaged with intervention components although the core elements represented minimum requirements for participation [46]. Control sites received no intervention except for training and support to collect study data.

### Outcomes

The primary outcome was a composite of the proportion of patients with suspected encephalitis whose care met both of the following criteria: a lumbar puncture performed within 12 hours of hospital admission unless clinically contraindicated; and intravenous aciclovir given within six hours of admission to hospital.

Secondary outcomes included the proportions of patients with suspected encephalitis who:

- Were started on intravenous aciclovir within an appropriate dosage range for a neurological presentation (adults and children analysed separately).
- Had a lumbar puncture performed within 12 hours of admission unless there was a clinical contraindication.
- Had a lumbar puncture at any point during the index presentation.
- Had either magnetic resonance imaging (MRI) or CT scan within 24 hours of admission.
- Had a lumbar puncture, who had the following CSF investigations performed: calculation of the plasma to CSF glucose ratio and having HSV polymerase chain reaction (PCR) performed.

### Data collection

We used retrospectively extracted pre-intervention data from case notes between 3^rd^ February 2013 and 2^nd^ February 2014 to provide data for the feedback intervention. We collected trial outcome data between 3^rd^ February 2014 and 2^nd^ February 2015 within the year following the launch of the implementation package.

Patients were identified during these two time periods using four methods; discharge code search for encephalitis, a microbiology search for patients who had had a lumbar puncture, patients who had received intravenous (IV) aciclovir for anything other than a dermatological condition and patients who had had either a CT head or MRI head. We used these combined approaches to maximise likelihood of case identification. The first two methods were compulsory at each site to reduce the likelihood of differences in case mix potentially resulting from different methods of detection confounding trial outcomes. We included both adult and paediatric cases, excluding neonates (up to four weeks) where management is different. We aimed to identify at least 20 cases per site with a maximum limit of 40 cases per site to preserve balance between sites. Given the large numbers of patients with records of CSF examination from all lumbar punctures, we asked local staff to order all cases by surname and date of birth before selecting every tenth case if there were less than 100 patients (i.e. a systematic 10% sample), and every twentieth case if there was over 100 patients (a systematic 5% sample), for eligibility screening. We used this approach to case identification to protect against post-randomisation selection bias.

Data were collected using structured case review forms. As no patient identifiable data were sent to the central trial team, we did not require patient consent as confirmed by the Declaration of Helsinki and the NHS Research Ethics Committee. We trained data collectors, mainly doctors who were undertaking speciality training and nurses, via face-to-face meetings and/or written briefing materials.

We collected data to assess time from admission to a range of investigations and interventions (e.g. lumbar puncture, aciclovir treatment and neuroimaging). We also collected data to assess the appropriateness of lumbar punctures and aciclovir dosages (contraindications, weight, and renal function). We reduced the number of items in the form following pre-intervention data collection to facilitate post-intervention data collection.

### Sample size

Using preliminary unpublished data from another Brain Infections UK study of 315 patients across 26 hospitals in four deaneries, we estimated the standard deviations of the deanery and hospital random effects to be 0.244 and 1.108, respectively, and the pre-intervention proportion of adherence to the primary outcome to be 5%. Assuming a 15% absolute increase in adherence to the primary outcome measure to 20% in intervention hospitals and identification of 20 cases in each of 24 hospitals, we calculated the required sample size to achieve at least 80% power, testing at the 5% level of significance, as follows.

Using the proposed analysis plan (see “Analysis” below) we simulated data under the stated assumptions, analysed the data using the glmer() function of the lme4 package in R [47] and calculated the p-value of the generalised likelihood ratio test of the null hypothesis of no treatment effect. We then repeated the simulation and used the observed proportion of simulated p-values less than 0.05 as an estimate of the power, stopping the simulations when the negligible Monte Carlo error became negligible. This gave the required sample size per hospital as 20 patients; to allow for possible under-recruitment in some of the smaller hospitals, we therefore sought to recruit 25 patients per hospital, but to close recruitment when we achieved a total of at least 560 patients.

### Randomisation

**Fig 1:**
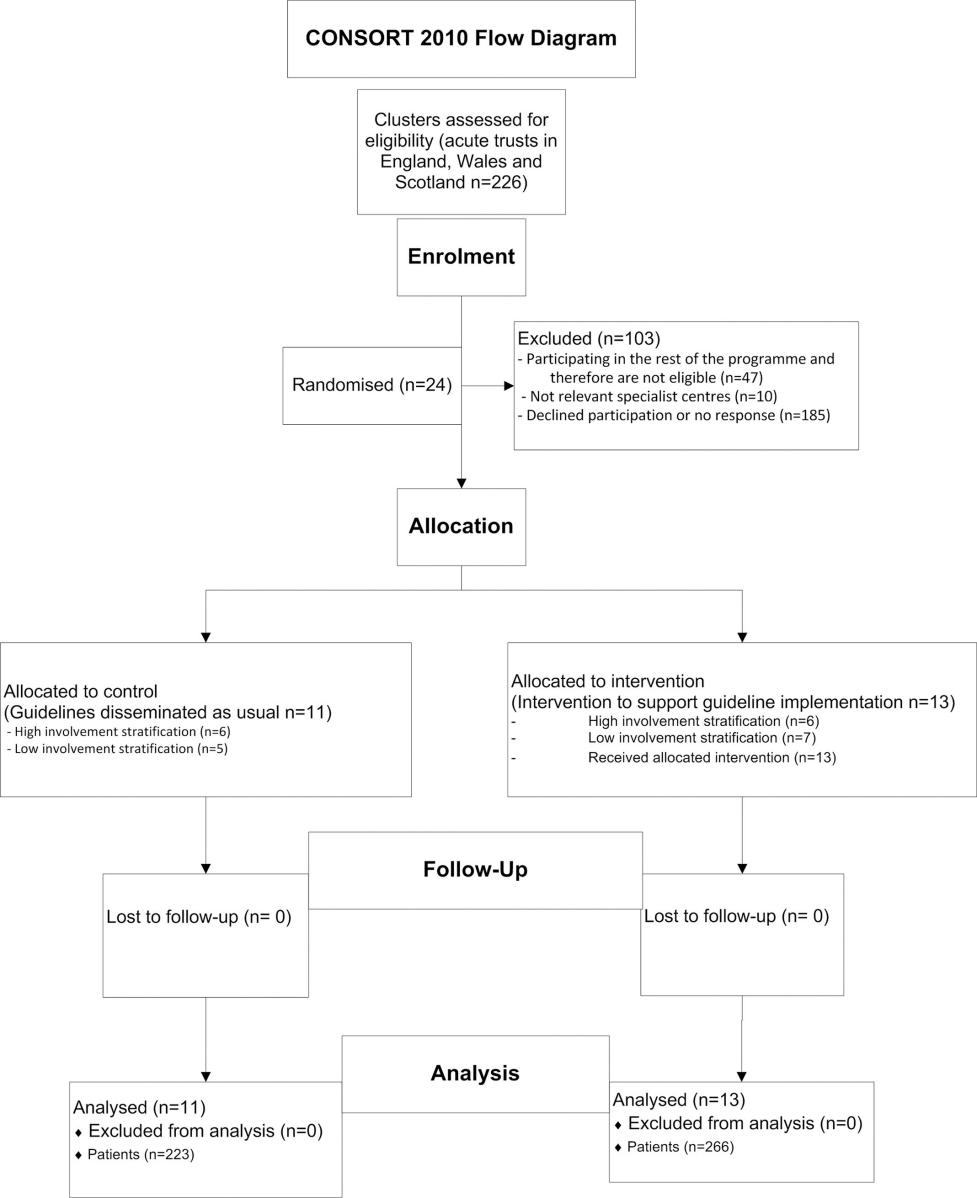
ENCEPH Cluster RCT CONSORT flow diagram. Fig 1 represents recruitment through both control and intervention arms in this study.

We used deaneries as the unit of randomisation to minimise contamination between hospitals within the same deanery. We defined two blocks of deaneries, a block of six where research teams were already actively involved in other ENCEPH UK studies prior to our RCT starting site recruitment, and a block of six where there were no such ongoing studies (Fig 1). No hospital was able to take part in this study if they were already involved in any other part of the ENCEPH UK programme. A statistician (PD) randomised equal numbers of clusters within each block to the intervention and routine arms, blinded to hospital identity. There was no concealment to randomisation allocation at the cluster level given the nature of the intervention.

### Analysis

We assessed primary and secondary outcomes as described above. We estimated the appropriateness of paediatric aciclovir treatment using 5^th^ and 95^th^ percentiles of estimated weight and length [48] when values were missing. Where values for adult weight were missing, we assumed weights of 50–70kg for women and 60–80kg for men to allow us to estimate appropriateness of aciclovir dosage in adults. A reduced dose was considered appropriate in patients with recorded renal impairment.

The primary analysis fitted a generalised linear mixed model with binomial errors, logistic link, fixed effects for treatment (control versus intervention) and block (high or low level of previous involvement for the deanery in the ENCEPH UK research programme), and random effects for deanery and hospital [35]. Formally, if *p*_*btdh*_ denotes the probability of a positive outcome (y = 1) in block *b*, treatment arm *t*, deanery *d* and hospital *h*, the model is that:

$$Log\left\{\frac{p_{btdh}}{1-b_{btdh}}\right\}=\alpha_{b}+\beta_{t}+U_{d}+V_{h1}$$

where the *U*_*d*_ are independent, Normally distributed with mean zero and variance $\sigma_{d}^{2}$ and the *V_h_* are independent, Normally distributed with mean zero and variance $\sigma_{h}^{2}$. The quantity of interest is the treatment effect, *β*_1_−*β*_0_, where *t* = 0 and *t* = 1 denote control and intervention, respectively.

We fitted the model using the glmer() function of the lme4 package in R [48]. Model fit was checked by examining scatterplots of residuals against fitted values.

We also compared outcomes between adults and children by adding this as a blocking factor within the statistical analysis. The statistician (PD) remained blind to randomisation allocation for the analysis.

### Patient involvement

A representative from the Encephalitis Society was involved in the study design with the particular remit of ensuring the research retained patient benefit as a primary objective, as well as providing subsequent opportunities for the study findings to be disseminated to a wider audience. Our intervention directly targeted healthcare professionals, aiming to help them overcome barriers to the timely and appropriate investigation and management of suspected encephalitis. However, as part of the intervention training the healthcare professionals received, a patient representative from the Encephalitis Society shared their experience of a non-timely diagnosis. This patient representative was an active member of the Programme Steering Committee and advised on the study design, outcome selection and conduct. The intervention was designed to promote best clinical practice, and thus there should have been no additional intervention burden to patients. Individual patient consent was not required as patient data were retrospectively collected by members of their own healthcare teams and anonymised.

### Ethical review

This project was approved by Preston North West Research Ethics Committee on 3^rd^ May 2013 (13/NW/0279).

## Results

We recruited a total of 24 hospitals from 12 of the 19 UK teaching deaneries. Following randomisation by deanery, there were 13 hospitals within six intervention deaneries and 11 hospitals within six control deaneries. A mix of large tertiary and smaller district general hospitals was recruited, broadly representing national provision. We identified 489 patients with suspected encephalitis, 266 in intervention hospitals and 223 in control hospitals. The number of hospitals per deanery ranged from one to four and number of cases from one to 38 (mean 20.4). The mean patient age was lower in intervention hospitals (45 years, standard deviation [SD] of 30) compared with controls (51 [27.6] years) as was the percentage of males (45.7% and 50% respectively; Table 1) demonstrating similar characteristics across both study arms. Case ascertainment was similar across both the control and intervention arms. The CONSORT diagram summarises recruitment, participation, and analysis (Fig 1 and S1 Table).

**Table 1:** Characteristics of hospital sites.

	Intervention	Control
**Number of hospitals**	13	11
**Number of patients recruited**	266	223
**Mean patient recruitment per site**	20.5	20.3
**Female**	133 (50%)	121 (54.3%)
**Average patient age (SD, range)**	45 (30, 0 to 100)	51 (27.6, 0 to 97)
**Number of adult patients**	206 (77.4%)	188 (84.3%)
** ‘Suspected encephalitis’ recorded in the notes**	37 (13.9%)	21 (9.4%)
** ‘Probable encephalitis’ (or equivalent) recorded in the notes**	47 (17.7%)	42 (18.8%)
**Number of patients identified using discharge codes**	86 (32.3%)	72 (32.3%)
**Number of patients identified through aciclovir usage**	39 (14.7%)	33 (14.8%)
**Number of patients identified through CSF examination**	132 (49.6%)	109 (48.9%)
**Number of patients identified through performance of a CT head scan**	9 (3.4%)	9 (4.0%)

Absolute numbers are displayed for all variables with percentages shown in brackets. Percentages were calculated using the patient variable against the number of patients within the arm.

There was marked variation between hospitals across both arms in adherence to the primary outcome (range 0% to 40%; interquartile range 4.71% to 21.74%). Across both intervention and control hospitals, overall pre-intervention adherence to the primary outcome was 8.5% (36 out of 422 patients) and post-intervention adherence was 14.1%.

Table 2 summarises crude estimates of the treatment effect for the primary and secondary outcomes without adjustment for clustering or covariate effects. Achievement of the primary outcome was 13.5% in intervention hospitals and 14.8% in controls.

**Table 2:** Crude trial primary and secondary outcomes unadjusted for clustering or covariate effects.

		Intervention			Control			
		Number eligible	Number meeting outcome	Percentage	Number eligible	Number meeting outcome	Percentage	Percentage difference (95% confidence interval)
**Primary outcome** **Lumbar puncture performed within 12 hours and IV aciclovir administered within 6 hour**	**Total**	266	36	13.53	223	33	14.80	-1.26 (-7.48, 4.95)
	**Adults**	206	25	12.14	190	29	15.26	-3.13 (-9.91, 3.66)
	**Paediatrics**	60	11	18.33	33	4	12.12	6.21 (-8.62, 21.04)
**Secondary outcomes:** **IV aciclovir administered within 6 hours**	**Total**	266	81	30.45	223	63	28.25	2.20 (-5.89, 10.29)
	**Adults**	206	52	25.24	190	52	27.37	-2.13 (-10.81, 6.56)
	**Paediatrics**	60	29	48.33	33	11	33.33	15.00 (-5.46, 35.46)
**Lumbar puncture performed within 12 hours unless there was a clinical contraindication**	**Total**	266	70	26.32	223	66	29.60	-3.28 (-11.27, 4.71)
	**Adults**	206	48	23.30	190	58	30.53	-7.23 (-15.96, 1.50)
	**Paediatrics**	60	22	36.67	33	8	24.24	12.42 (-6.61, 31.46)
**Lumbar puncture at any time**	**Total**	266	234	87.97	223	209	93.72	-5.75 (-10.79, -0.71)
	**Adults**	206	180	87.38	190	178	93.68	-6.31 (-12.01, -0.60)
	**Paediatrics**	60	54	90.00	33	31	93.94	-3.94 (-15.07, 7.19)
**Lumbar puncture with CSF/serum glucose ratio calculated**	**Total**	266	86	32.33	223	78	35.00	-2.65 (-11.06, 5.77)
	**Adults**	206	65	31.55	190	65	34.21	-2.66 (-11.92, 6.60)
	**Paediatrics**	60	21	35.00	33	13	39.39	-4.39 (-24.98, 16.19)
**Lumbar puncture and a sample taken for HSV PCR**	**Total**	266	182	68.42	223	175	78.48	-10.05 (-17.82, -2.29)
	**Adults**	206	141	68.45	190	147	77.37	-8.92 (-17.62, -0.22)
	**Paediatrics**	60	41	68.33	33	28	84.85	-16.52 (-33.49, 0.46)
**CT or MRI within 24 hours**	**Total**	266	153	57.52	223	120	53.81	3.71 (-5.13, 12.54)
	**Adults**	206	129	62.62	190	112	58.95	3.67 (-5.95, 13.30)
	**Paediatrics**	60	24	40.00	33	8	24.24	15.76 (-3.41, 34.93)
**Appropriate dose of aciclovir administered**	**Total**	266	136	51.13	223	131	58.74	-7.62 (-16.44, 1.21)
	**Adults**	206	122	59.22	190	119	62.63	-3.41 (-13.02, 6.20)
	**Paediatrics**	60	14	23.33	33	12	36.36	-13.03 (-32.62, 6.56)

Primary and secondary outcomes for control and intervention arms across all adults and paediatric patients. Number eligible shows denominator.

Modelling indicated no significant difference between intervention and control hospitals for the primary outcome of a lumbar puncture performed within 12 hours of hospital admission and aciclovir given within 6 hours (estimated treatment effect -0.188 with standard error 0.377; p = 0.619; 95% confidence interval [CI] -0:927, 0.552). There was also no significant intervention effect for any of the seven secondary outcomes (Table 3). For the seventh secondary outcome of appropriate dosing of aciclovir administration, although across the whole sample, children were significantly less likely than adults to be prescribed correct doses of aciclovir (estimated effect -1.073 with standard error 0.412; p = 0.009; Table 4). There was no significant interaction between intervention and age group for this nor or any other secondary outcome (Table 5).

**Table 3:** Maximum likelihood estimates of intervention effect for secondary outcomes.

Secondary outcome	Estimate	Standard error	p-value
Lumbar puncture performed within 12 hours unless there was a clinical contraindication	*- 0*.*11*	0.30	0.73
Lumbar puncture at any time	*- 0*.*75*	0.43	0.08
CT or MRI within 24 hours	*0*.*17*	0.38	0.66
Lumbar puncture with CSF/serum glucose ratio calculated	*0*.*12*	0.43	0.78
Lumbar puncture and a sample taken for HSV PCR	*0*.*16*	0.70	0.82
**IV aciclovir administered within 6 hours**	***0*.*10***	**0.36**	**0.78**

**Table 4:** Estimates of fixed effects in the model for the secondary outcome, correct administration of aciclovir.

Parameter	Estimate	Standard error	p-value
Intercept	*0*.*68*	0.19	0.00
Block	*-0*.*37*	0.21	0.74
Treatment	*-0*.*12*	0.23	0.60
Age group	*-1*.*07*	0.41	0.01
**Interaction**	***-0*.*60***	**0.54**	**0.26**

**Table 5:** Maximum likelihood estimates of interaction effect between treatment and age group for secondary outcomes.

**Secondary outcome**	**Estimate**	**Standard error**	**p-value**
Lumbar puncture performed within 12 hours unless there was a clinical contraindication	*0*.*79*	0.62	0.21
Lumbar puncture at any time	*0*.*10*	1.01	0.92
CT or MRI within 24 hours	*0*.*42*	0.62	0.49
Lumbar puncture with CSF/serum glucose ratio calculated	*-0*.*42*	0.69	0.54
Lumbar puncture and a sample taken for HSV PCR	*-0*.*62*	1.18	0.60
**IV aciclovir administered within 6 hours**	***0*.*12***	**0.36**	**0.74**

Estimated variances of the random effects for deanery and hospital were 2.73x10^-5^ and 0.18, respectively. These were substantially smaller than we had anticipated, possibly because they had been estimated from an earlier dataset without covariates, whereas we were able to adjust for the effects of covariates in our actual analysis.

## Discussion

A multifaceted implementation strategy based on identified barriers to change had no effect on the initial hospital management of patients with suspected encephalitis. Overall adherence to recommended practice remained low despite evidence of a modest overall improvement across both intervention and control sites compared with the pre-intervention period. Although most patients had a lumbar puncture at some point, less than a third had a lumbar puncture within 12 hours of admission. Furthermore, less than a third were prescribed aciclovir within six hours of admission. There are still major challenges facing any drive to improve initial care for suspected encephalitis. Our evaluation represents a clear advance on previous intervention studies targeting suspected encephalitis, improving validity by use of a randomised design and generalisability by including multiple sites across the UK [1,3,49]. Our theory-informed implementation strategy was tailored according to identified needs and barriers and based upon interventions and resources typically available to improve quality of care [30].

There were five main study limitations. First, there was a threat to internal validity of post-randomisation selection bias, whereby greater awareness of the trial amongst intervention compared with control hospitals could have influenced identification of cases of suspected encephalitis and differentially affected case mix and attainment of study outcomes. We standardised and maximised case identification by combining methods. The mean number of suspected cases identified per site, methods of identification and patient demographic factors were similar between intervention and control site. Second, retrospective data collection depended upon the quality of routine clinical recording, which was variable. There was a consistent lack of recording of patient weight upon admission across both trial arms. We therefore used estimates and assumptions to assess appropriateness of aciclovir dosage in adults and children. Third, following initial training and provision of materials, much intervention delivery was delegated to clinicians in each hospital; this is likely to have resulted in variable fidelity of intervention delivery. However, within a pragmatic trial, the implementation strategy reflected what could reasonably be achieved within limited national and local resources [46]. Fourth, our composite outcome measure may have set the bar too high, so that both performing lumbar punctures and prescribing aciclovir within limited time periods might have been too difficult to achieve within busy emergency care settings. However, we found no differences in any of the other less time-contingent secondary outcomes, suggesting that our primary outcome was not too strict. Fifth, our negative findings, as is generally the case, may yet be explained by a lack of statistical power. Basing our sample size upon an absolute increase of 15%, from 5% to 20%, in adherence to the primary outcome looks over-optimistic in hindsight. However, our estimates of effect size were within the ranges of those typically reported for other implementation trials [31], we had selected a primary endpoint with low baseline performance and hence considerable scope for improvement, and we had hoped to leverage additional effects by tailoring our intervention to identified barriers. Again, the absence of any signals suggesting an intervention benefit across any of the outcomes use indicates that a lack of statistical power is unlikely to explain our negative findings.

Our multifaceted implementation strategy was designed to address multiple barriers to the recommended management of suspected encephalitis. We provide further evidence that multifaceted strategies cannot be assumed to be more effective than single interventions although there is a lack of head-to-head comparisons and a risk of confounding by indication (i.e. investigators may select multiple interventions to address more challenging implementation problems).[50, 51] We also show that one approach to theory-guided, intervention tailoring did not work for this targeted problem and context.

One possible explanation for the lack of any intervention effect is that any modest intervention effects may have been overshadowed by wider improving trends in the management of patients with suspected encephalitis. Adherence to several outcomes, including time to lumbar puncture and time to aciclovir, in both trial arms was comparable or better than earlier studies [1–3,5,9,11–13,18–21,49]. We also observed an improvement in the primary outcome from 8.5% to 14.1% across all intervention and control hospitals over the study period; although this could partly be explained by changes we made following pre-intervention data collection to facilitate case identification. Furthermore, our implementation strategy aimed to deliver improvements over and above existing trends.

The most plausible explanation for the lack of intervention effect is that, coupled with variable fidelity of delivery, our intervention simply failed to target the key determinants of adherence to recommended practice and overcome multiple barriers and competing priorities within pressurised acute care settings. Other major and better resourced improvement initiatives targeting hospital care have also failed to translate into improved frontline patient care [52,53]. Our trial findings are a pertinent reminder of the need for rigorous, controlled evaluations of quality improvement strategies [54].

The majority of implementation studies have generally targeted the care of more prevalent, long-term conditions [30]. Although the presentation of suspected encephalitis as opposed to confirmed encephalitis is not uncommon, it is rarer compared with other emergencies, such as suspected stroke. It is likely that a more sustained or different type of approach may be necessary to embed recommended initial management into clinician cognitions and routines [51]. Nevertheless, it is still notable that there was some divergence between paediatric and adult outcomes, with non-significant trends towards timely lumbar puncture and aciclovir administration in intervention hospitals according to crude effect estimates. Our trial was insufficiently powered to detect any such sub-group effects but it may be the case that our intervention might have had greater leverage within the context of acute paediatric care, where there is a greater emphasis on the rapid detection and treatment of suspected CNS infections [5].

There is a growing evidence base on the effects of implementation strategies. For example, the relatively modest effects of audit and feedback can be enhanced by ensuring feedback is delivered in both written and verbal formats, is provided more than once, and includes both explicit targets and an action plan [55]. There are therefore opportunities to strengthen the content and delivery of implementation strategies [56], although any subsequent benefits need to be weighed up against the increased costs of more intensive strategies [57].

## Conclusion

Clinicians struggle to achieve timely diagnosis and management of encephalitis in hospital emergency settings, leading to worse patient outcomes. A randomised trial of an implementation strategy tailored to identified barriers and enablers and comprising a range of interventions which could reasonably be delivered within available resources for quality improvement had no effect on patient care, though there were signals of improved management for children. The failure to show an effect in the intervention hospitals may partly be because of generally improved management over time in all hospitals. Different approaches, such as targeting by type of clinical presentation rather than by specific condition, need to be developed and evaluated to improve the care of suspected encephalitis and similar challenging clinical presentations.

## Supporting information

S1 Table CONSORT 2010 checklist of information to include when reporting a cluster randomised trial. (DOCX)

S1 File Cluster RCT protocol V6.0. Protocol approved by NHS Research Ethics Committee prior to start of trial recruitment.
(PDF)

S2 File Cluster RCT protocol V2.0. Initial Protocol approved by NHS Research Ethics Committee.
(PDF)

S3 File The study data are available in the supporting information Files (Backman, RCT data 161018 for PONE). (XLSX)
