# Supplementary material for: A pragmatic cluster randomised controlled trial of a tailored intervention to improve the initial management of suspected encephalitis
Source: PLoS One. 2018 Dec 6;13(12):e0202257. doi: 10.1371/journal.pone.0202257 (PMC6283633; doi:10.1371/journal.pone.0202257)
Supplement: S2 File — Initial Protocol approved by NHS Research Ethics Committee. (PDF) [file pone.0202257.s003.pdf]

ENCEPH UK
Intervention RCT

Development and Evaluation of an Intervention Based Around the National Guidelines on the Management of Suspected Encephalitis, and its evaluation through a Cost Effectiveness Analysis

ENCEPH UK – Intervention Randomised Controlled Trial

Version 2.0 260313

Identifying Numbers: (ISRCTN) TBC
NIHR (Funder) Reference: PR-PG-108-048

General Information

This document describes the ENCEPH UK Intervention Cluster Randomised Controlled Trial (RCT) and provides information about procedures for entering patients into it. The protocol should not be used as an aide-memoire. Every care was taken in its drafting, but corrections or amendments may be necessary. These will be circulated to the registered investigators in the trial, but centres entering patients for the first time are advised to contact the coordinating centre (Brain Infection Group, Liverpool) to confirm they have the most up to date version. Clinical problems relating to this trial should be referred to the relevant Chief Investigator via the relevant study team personnel in the Brain Infections Group.

Statement of Compliance

This study will be carried out in accordance within the Research Governance Framework and follow the International Conference on Harmonisation Good Clinical Practice.

Contact Details: Institutions

Sponsor 1 (University of Liverpool):	Sponsor 2 (TBC):	
Alex Astor<br>University of Liverpool<br>Research Support Office<br>2nd Floor, Block D<br>Waterhouse Building<br>Liverpool<br>L69 3BX<br>Tel 0151 794 8739<br>sponsor@liv.ac.uk	Malcolm Steiger<br>The Walton Centre for Neurology and Neurosurgery,<br>Lower Lane,<br>Fazakerley,<br>Liverpool<br>L9 7LJ<br>Tel 0151 525 3611	

Contact Details: Individuals

Individual Authorised to Sign the Protocol and Protocol Amendments on behalf of the Sponsor:	Chief Investigator (CI):	Programme Manager
Alex Astor<br>University of Liverpool<br>Research Support Office<br>2nd Floor, Block D<br>Waterhouse Building<br>Liverpool<br>L69 3BX<br>Tel 0151 794 8722<br>sponsor@liv.ac.uk	Professor Tom Solomon<br>Institute of Infection and Global Health<br>Ronald Ross Building<br>University of Liverpool<br>8 West Derby Street<br>Liverpool<br>L69 7BE<br>tsolomon@liv.ac.uk	Hayley Jelleyman<br>Institute of Infection and Global Health<br>Ronald Ross Building<br>University of Liverpool<br>8 West Derby Street<br>Liverpool<br>L69 7BE<br>h.e.jelleyman@liverpool.ac.uk
**Programme Clinical Research Fellow**	**Trial Coordinator:**	**Trial Statistician:**
Dr Sylviane Defres<br>Institute of Infection and Global Health<br>Ronald Ross Building<br>University of Liverpool<br>8 West Derby Street<br>Liverpool<br>L69 7BE<br>sdefres@liverpool.ac.uk	Dr Ruth Backman<br>Institute of Infection and Global Health<br>Ronald Ross Building<br>University of Liverpool<br>8 West Derby Street<br>Liverpool<br>L69 7BE<br>r.backman@liverpool.ac.uk	Professor Peter Diggle<br>Department of Epidemiology and Population Health,<br>University of Liverpool<br>Leahurst Campus<br>Chester High Road<br>Neston<br>Cheshire<br>CH64 7TE<br>p.diggle@lancaster.ac.uk
**Data Manager:**	**Co-Investigator**	**NIHR Clinical Research Fellow**
Chloe Smith<br>Institute of Infection and Global Health<br>Ronald Ross Building<br>University of Liverpool<br>8 West Derby Street<br>Liverpool<br>L69 7BE<br>Chloe.Smith@liverpool.ac.uk	Prof Robbie Foy<br>Professor of Primary Care<br>Leeds Institute of Health Sciences<br>University of Leeds<br>Charles Thackrah Building<br>101 Clarendon Road<br>Leeds LS2 9LJ<br>T: +44 (0) 113 343 4879<br>r.foy@leeds.ac.uk	Dr Benedict Michael<br>Institute of Infection and Global Health<br>Ronald Ross Building<br>University of Liverpool<br>8 West Derby Street<br>Liverpool<br>L69 7BE<br>Benedict.Micheal@liverpool.ac.uk

Contact Details: Individuals

**Health Economics**<br>Dr Antonieta Medina Lara<br>Senior Lecturer in Health Economics<br>Health Economics Group<br>Penninsula College of Medicine & Dentistry<br>Exeter University<br>Veysey Building<br>Salmon Pool Lane<br>Exeter<br>EX2 4SG<br>A.Medina-Lara@exeter.ac.uk	**Intervention development**<br>Dr Ciara Kierans<br>Institute of Psychology, Health and Society<br>University of Liverpool<br>3rd Floor, Whelan Building<br>Quadrangle<br>Brownlow Hill<br>Liverpool<br>L69 3GB<br>C.Kierans@liverpool.ac.uk	

Table of Contents

1 Protocol Summary 8
2 Background Information 11
2.1 Introduction 11
2.2 Objectives 11
3 Selection of Centres/Clinicians 12
3.1 Centre/Clinician Inclusion Criteria 12
3.2 Centre/Clinician Exclusion Criteria 12
4 Trial design 12
5 Study Population 14
5.1 Inclusion Criteria 14
5.2 Exclusion Criteria 15
5.3 Patient Transfer and Withdrawal 15
6 Intervention developement and delivery 15
7 Assessments and Procedures 18
7.1 Data collection 18
7.2 Other Assessments 19
7.3 Trial Closure 19
8 Statistical Considerations 20
8.1 Randomisation 20
8.2 Outcome Measures 20
8.3 Sample Size and Analysis 20
9 Ethical Considerations 21
9.1 Ethical Considerations 21
9.2 Ethical Approval 21
10 Trial Monitoring 22
10.1 Risk Assessment 22
10.2 Source Documents 22
10.3 Data Capture Methods 22
10.4 Data checks 24
10.5 Records Retention 25
11 Indemnity 26
12 Financial Arrangements 27
13 Trial Committees 27
13.1 Programme Steering Group 27
13.2 Programme Management Group 27
13.3 Study Management Group 27
14 Publication 28
15 Protocol Amendments 29
15.1 Version 1 (dd/mon/yyyy) 29
16 References 30

Glossary

CI	Chief Investigator
CNS	Central Nervous System
CRF	Case Report Form
CSF	Cerebrospinal Fluid
CT	Computer Tomography
EEG	Electroencephalogram
HPA	Health Protection Agency
HSV	Herpes Simplex Virus
ICERS	Incremental Cost-Effectiveness Ratio
ICH GCP	International Conference on Harmonisation Good Clinical Practice
ID	Identity
ISRCTN	International Standard Randomised Controlled Trial Number
IV	Intravenous
LP	Lumbar Puncture
MREC	Multi-centre Research Ethics Committee
MRI	Magnetic Resonance Imaging
N/A	Not Applicable
N/D	Not Done
NHS	National Health Service
NIHR	National Institute of Health Research
PI	Principal Investigator
PCR	Polymerase Chain Reaction
QALY	Quality Adjusted Life Year
QC	Quality Control
R&D	Research & Development
RCT	Randomised Controlled Trial
SSA	Site Specific Assessment

1 PROTOCOL SUMMARY

Title: Development and evaluation of an intervention based around the National guidelines on the management of suspected encephalitis, and its evaluation through a cost effectiveness analysis.

Population: Secondary care hospitals in the UK will be included. Hospital sites already participating in the ENCEPH-UK study will be excluded so as to reduce the risk of attention bias.

Number of Sites: Twenty eight secondary care hospitals will be recruited across the UK.

Study Duration: Two years per site (to include delivery of the intervention over four months and an evaluation at 12 months).

Description of Intervention: The intervention will target clinicians (and other healthcare workers) within the hospitals. Outcome data will be collected on patients (adults and children) with suspected or confirmed encephalitis.

Objectives:

1. To evaluate the effects of an intervention to improve the clinical management of suspected encephalitis in secondary care.
2. To evaluate the cost-effectiveness of the intervention.

<u>Primary outcome</u>

The proportion of patients with suspected encephalitis* whose care met all of the following criteria:

- Aciclovir given within 6 hours at the appropriate dose unless there was an alternative diagnosis;
- A lumbar puncture (LP) was performed within 12 hours of hospital arrival unless clinically contraindicated; and

- If LP performed at any time, the cerebrospinal fluid (CSF) was tested for herpes simplex virus (HSV) polymerase chain reaction (PCR), and CSF to serum glucose ratio determined.

The secondary outcomes will be:

- The proportion of all adults given intra venous (IV) aciclovir for a neurological presentation who met the definition of suspected encephalitis.
- The proportion of all children given IV aciclovir for a neurological presentation who met the definition of suspected encephalitis.
- The proportion of patients with suspected encephalitis who had a lumbar puncture performed within 12 hours unless there was a clinical contraindication.
- The proportion of patients with suspected encephalitis who had a lumbar puncture after resolution of a clinical contraindication to that LP.
- The proportion of patients with suspected encephalitis who had either Magnetic Resonance Imaging (MRI) or Computer Tomography (CT) scan within 24 hours of admission.
- For patients with HSV encephalitis, the proportion who die, have sequelae and appear to make a full recovery upon discharge.
- The proportion of patients with suspected encephalitis having had a lumbar puncture, who had the following CSF investigations performed:
    - CSF:serum glucose ratio calculated
    - HSV PCR performed
- An evaluation of the primary outcomes comparing adults and children enrolled in the RCT.
- A cost effectiveness evaluation of the intervention.

* Suspected encephalitis is defined as febrile illness or history of a febrile illness with altered behaviours, cognition, personality, or consciousness or new seizures or new focal neurological signs (please see section 5.1 for details).

Protocol Summary

Design

Two arm cluster randomised controlled trial comparing a guideline implementation intervention with routine practice.

Schematic of Study Design:

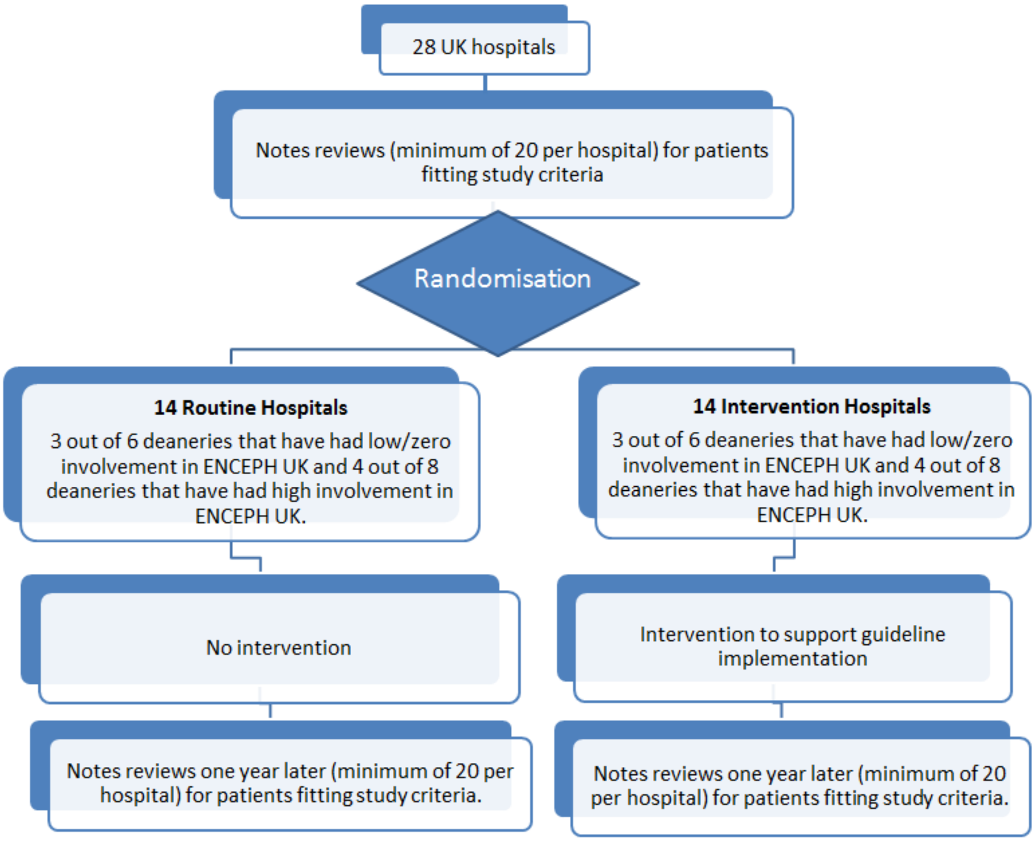

2 BACKGROUND INFORMATION

Overview

This study will evaluate the clinical effectiveness and cost-effectiveness of an intervention to support the implementation of national encephalitis guidelines. Intervention development will be informed by existing evidence on professional behaviour change and sub-studies within the ENCEPH UK programme. The intervention will target clinicians responsible for the diagnosis and initial management of suspected encephalitis. The use of a cluster randomised controlled trial (RCT) in twenty eight hospitals with an evaluation period of 12 months will allow both cost effectiveness and clinical outcomes to be assessed.

2.1 Introduction

Encephalitis (inflammation of the brain tissue) is one of the most important types of brain infection because of the devastating impact on those affected. It has a disproportionately large burden on the NHS and community through high health and economic costs, but this has never been quantified. In terms of research, encephalitis is relatively neglected because it is not easily funded from other sources, e.g. major charities.

HSV is the most common viral cause (1) and is treatable with aciclovir, but delays in treatment are associated with poor outcome. There has been little research into how and why delays occur. There are not economic studies in the UK that have attempted to estimate the economic impact of encephalitis. However, settlements for negligent management of a single encephalitis case are around £1-2 million.

2.2 Objectives

It has been found that many patients with suspected encephalitis are started late with aciclovir treatment. The barriers and enablers for the diagnosis and management of encephalitis patients will be assessed as part of a sub-study in the ENCEPH UK programme. This consists of semi structured interviews with health care professionals who have a variety of roles within several hospitals. From this, the major barriers and enablers to using the encephalitis guidelines will be deduced and a novel intervention will be developed and implemented in 14 hospitals over four months. This intervention will then be assessed for both clinical and cost effectiveness.

3 SELECTION OF CENTRES/CLINICIANS

Study centres will be initiated once all global (e.g. local research and development (R&D) approval) and study specific conditions (e.g. training requirements) have been met, and all necessary documents have been returned to the Brain Infections UK coordinating centre.

All centres will have a consultant and all centres must be equipped with the ability to perform, or have access to, CT/MRI scans and have aseptic conditions for conducting a LP to be performed. Hospitals will be randomised as a unit to either the standard care or intervention arm.

3.1 Centre/Clinician Inclusion Criteria

a. Local R&D approval
b. Completion and return of ‘signature and delegation log’ to Brain Infections UK
c. CV and Good Clinical Practice (GCP) certificate to accompany all research personnel recorded on the ‘signature and delegation’ log
d. Contractual agreements signed between site and study Sponsor
e. Receipt of evidence of completion (a) – (e) by Brain Infections UK

3.2 Centre/Clinician Exclusion Criteria

a. Not meeting the inclusion criteria listed above
b. Participating in ENCEPH UK Cohort studies

4 TRIAL DESIGN

A cluster RCT design has been chosen to compare routine practice with an intervention promoting ‘best practice’ within 28 hospitals. The intervention taking place in 14 hospitals will target clinicians. Data on patient care will be collected to assess clinical practice. It is not envisaged that there will be any study related risks to the patients as data collection methods will be similar to the clinical audit that have been performed over the last ten years within our group (2,3). Identifiable data will only be seen by a member of NHS staff currently working at the site and will not be sent to Brain Infections UK. The number of cases, management and cost effectiveness of the intervention will be assessed in all sites.

Primary outcome

The proportion of patients with suspected encephalitis* whose care met all of the following criteria:

- Aciclovir given within 6 hours at the appropriate dose unless there was an alternative diagnosis;
- A LP was performed within 12 hours of hospital arrival unless clinically contraindicated; and
- If LP performed at any time, the CSF was tested for HSV PCR, and CSF to serum glucose ratio determined.

The secondary outcomes will be:

- The proportion of all adults given intra venous (IV) aciclovir for a neurological presentation who met the definition of suspected encephalitis.
- The proportion of all children given IV aciclovir for a neurological presentation who met the definition of suspected encephalitis.
- The proportion of patients with suspected encephalitis who had a lumbar puncture performed within 12 hours unless there was a clinical contraindication.
- The proportion of patients with suspected encephalitis who had a lumbar puncture after resolution of a clinical contraindication to that LP.
- The proportion of patients with suspected encephalitis who had either MRI or CT scan within 24 hours of admission.
- For patients with HSV encephalitis, the proportion who die, have sequelae and appear to make a full recovery upon discharge.
- The proportion of patients with suspected encephalitis having had a lumbar puncture, who had the following CSF investigations performed:
    - CSF:serum glucose ratio calculated
    - HSV PCR performed
- An evaluation of the primary outcomes comparing adults and children enrolled in the RCT.
- A cost effectiveness evaluation of the intervention.

** Suspected encephalitis is defined as febrile illness or history of a febrile illness with altered behaviours, cognition, personality, or consciousness or new seizures or new focal neurological signs (please see section 5.1 for details).*

There will be a time to event analysis for all of the primary and secondary outcomes listed above.

5 STUDY POPULATION

5.1 Inclusion Criteria

Within this study, any patients who have suspected encephalitis within the twenty eight sites will be included. The inclusion criteria for suspected encephalitis are:

Patients with suspected encephalitis

(a) Mandatory

Acute or sub acute (<4 weeks) alteration in consciousness, cognition, personality or behaviour persisting for more than 24 hours. Personality / behaviour change includes: agitation, psychosis, somnolence, insomnia, catatonia, mood liability, altered sleep pattern and (in children): new onset enuresis, or irritability.

Plus ANY two of:

- Fever (> 38ºC) / Prodromal illness – acute or sub-acute
- Seizures: New onset
- Focal Neurological Signs – Acute or Sub-acute onset. These include
  - Focal weakness
  - Oromotor dysfunction
  - Movement disorders (chorea, athetosis, dystonia, hemiballisms, stereotypies, orolingual dyskinesia and tics) including Parkinsonism (bradykinesia, tremor, rigidity and postural instability)
  - Amnesia
- Pleocytosis: Cerbrospinal fluid white cell count >4 cells/ul
- Neuroimaging: Compatible with encephalitis
- Electroencephalogram (EEG): compatible with encephalitis

OR

(b) Clinical suspicion of encephalitis but above investigations have not yet been completed

OR

(c) Clinical suspicion of encephalitis and the patient died before investigations completed

5.2 Exclusion Criteria

- Patients with non-infectious Central Nervous System (CNS) disorders due to hypoxic, ischemic, vascular, toxic and metabolic causes

5.3 Patient Transfer and Withdrawal

There is a possibility that patients could be transferred to and from the sites within this study. Efforts will be made to establish if any centres regularly receive patient transfers and appropriate recording mechanisms will be put in place. The first 24 hours of a patient's management are the most critical for the primary outcome. Therefore, the patient will be counted at the site of their first admission if initial clinical management takes place within this time period. Should a patient be transferred to a different site within 12 hours, there should still be sufficient data to count the patient within that original study site.

6 INTERVENTION DEVELOPEMENT AND DELIVERY

Intervention development involves a number of steps, some of which have been undertaken during earlier studies in the programme. These steps comprise:

- Defining priorities for implementation, i.e. key guideline recommendations where there is scope for improved clinical performance;
- Identifying perceived barriers to and enablers of recommended practice;
- Defining candidate intervention components; and
- Constructing a coherent intervention package.

Defining priorities for implementation. We already have a good understanding of hospital based performance from studies recently conducted (4, 5), as well as preliminary analysis of the Health Protection Agency (HPA) cohort study. During the ENCEPH-UK programme, we will continue to collect data on the timing of the various diagnostic and treatment decisions.

One priority will be to increase the proportion of patients with suspected encephalitis appropriately and promptly started on treatment with aciclovir. This is chosen because of compelling evidence that not starting treatment for 48 hours or more is associated with a poor outcome in patients with HSV encephalitis.

Other priorities for attention will include the diagnostic tests performed, their sequence and timing, and the grade of clinicians involved in decision making, looking in particular for major deviations away from the guidelines. These are defined as those serious enough to cause appreciable morbidity and mortality (6), and are based on published guidelines on the management of suspected CNS infections (7-11):

- Performing a LP, without performing imaging first, in a patient with contraindications to an immediate LP (8, 10);
- Delaying a LP in order to perform imaging, when there is no contraindication to an immediate LP (8, 10, 11);
- Failing to perform a diagnostic LP in a patient with a suspected CNS infection, and no contraindications (7, 8, 10, 11); and
- Failure to investigate CSF for cell count, protein, and CSF to plasma glucose ratio when performing a LP.

Priorities for implementation (i.e. key guideline recommendations) will be derived from the Association of British Neurologists and British Infection Association National Guidelines for Management of Suspected Encephalitis (12).

Identifying perceived barriers to and enablers of recommended practice. Ideally, any intervention to change a given professional behaviour should address factors known to affect that behaviour. For example, if a lack of skills is deterring doctors from performing timely, indicated lumbar punctures, then the intervention could include some form of training in performing lumbar punctures. Often, multiple factors affect professional behaviour and more than one approach may be needed to help change clinical behaviour. For example, lack of confidence (self-efficacy) might also be a factor in deterring doctors from performing indicated lumbar punctures. In that case, some form of clinician mentoring which supports doctors in performing lumbar punctures in relatively easy circumstances or patients until they have mastered the technique in more challenging circumstances or patients might be appropriate.
This work will have been undertaken during the cross sectional interview telephone survey of health care staff undertaken during the Prospective Cohort Study.

Defining candidate intervention components. Interventions can be envisaged as having two types of component: a behaviour change technique and a method of delivering that technique. In the above example, a graded task approach is a behaviour change technique, involving the practice of a procedure through increasingly levels of difficulty until it is mastered, which may help doctors in performing lumbar punctures more routinely. The behaviour change technique is delivered through clinician mentoring. Sometimes, a behaviour change technique can be delivered in more than one way. For example, persuasive communication to increase motivation to perform timely lumbar punctures could be delivered via an interactive educational session or through computerised prompts.

Constructing a coherent intervention package. The choice of any given intervention delivery method can be driven by a number of considerations. These include: feasibility (e.g. whether it is possible to adapt local computerised clinical systems to incorporate automated prompts and reminders); acceptability (e.g. to local clinical leaders and their staff); cost (e.g. so that the costs of the intervention do not outweigh the likely benefits); and sustainability (e.g. can the intervention be embedded within continuing systems and routines).

Table 1 illustrates some hypothetical ways in which interventions might be constructed by linking different determinants of behaviour to behaviour change techniques and intervention delivery methods. This phase of development therefore involves judgments about how best to integrate an intervention package which can be delivered to the hospitals randomized to the intervention arm. The intervention will be delivered to the intervention hospitals during a four month period and there may be a short reminder every four months to ensure that staff that have newly rotated to that hospital are aware of the intervention if applicable.

Table 1. Illustrative examples of intervention construction

**Clinical behaviour targeted**	Performing an immediate lumbar puncture in a patient without contraindications	Investigating CSF for cell count, protein and CSF to plasma glucose ratio	Starting treatment with aciclovir within 6 hours in patients with suspected encephalitis
**Determinant of behaviour targeted (domain from interviews)**	Lack of knowledge of relevant clinical history and signs (knowledge)	Inability to recall all required investigations (memory, attention and decision processes)	Erroneous beliefs about consequences of not starting treatment (beliefs about consequences); not perceived as standard practice (social influences)
**Behaviour change technique(s)**	Awareness raising	Provision of prompts, triggers and cues	Persuasive communication; social pressure and modelling
**Intervention delivery method(s)**	Educational meetings; printed educational materials	(Easily available) departmental protocols and computerised pathways; prompts in electronic or paper forms for ordering investigations	Educational meetings outlining evidence base and incorporating patient narratives to illustrate consequences of delayed treatment; demonstration of appropriate use by opinion leader

7 ASSESSMENTS AND PROCEDURES

7.1 Data collection

Data will be collected by trained research nurses. The data will be pseudo-anonymised during the completion of the case report form (CRF) with the study number acting as the encryption key (please see below for details). Data will be collected for the previous 12 months and then again in 12 months' time.

7.2 Other Assessments

Cost-effectiveness analysis

Methods. Quantities of resource utilisation will be collected from trial records, while unit costs will be obtained from publicly available data published routinely. Costs related to inputs associated with health care services as used in the trial will be estimated at its conclusion. Costs and health benefits occurring after 12 months will be discounted at 3.5% per annum. Therefore, results will include, in addition to incremental costs and benefits, an estimate of total cost per patient for each arm. In order to estimate Quality Adjusted Life Years (QALYs), utility values will be estimated from the results of administering the SF-36 and EQ-5D in the Quality of Life sub-study, following Brazier's methodology (13). Estimates of costs and health outcome (utilities) will be used to populate a model of encephalitis patient management under the status quo and the alternative under new guidelines being adopted. The model will cover the remaining patient lifetime within which the (probability of) cost-effectiveness of the alternative will be analysed (14).

Statistical Methods. Cost data will be analysed using regression methods for handling censored cost data. Costs and benefits will be analysed jointly using a bivariate probability distribution. Sample uncertainty in estimated cost difference and Incremental Cost-Effectiveness Ratios (ICERs) between arms groups will be described using bootstrapped confidence intervals (15). Data analysis will be performed using a suitable statistical package such as R or STATA.

Uncertainty Analysis of Decision Model Results. Parameter uncertainty will be accounted for through univariate sensitivity analysis and probabilistic sensitivity analysis.

7.3 Trial Closure

The site shall be deemed as closed after the data at 12 months has been completed and all data queries resolved. When this has happened at all sites the database will be locked.

8 STATISTICAL CONSIDERATIONS

8.1 Randomisation

To ensure the best quality data within this study control and intervention sites must not have any routine crossover of staff between them, for example, trainee doctors moving as part of the standard rotation pattern within their training deanery. Therefore, we will take deanery boundaries into account during recruitment and randomise using the deaneries as randomisation units. We intend to include a range of types of hospital in the trial to improve generalisability. The mix of these should broadly represent national provision. We will use deanery as the unit of randomisation, to guard against leakage of information between hospitals within the same deanery as trainee doctors move between hospitals. We will define two blocks of deaneries, a block of 8 in which research teams are currently actively involved in the ENCEPH UK programme and a block of 6 in which research teams are not currently actively involved in the ENCEPH UK programme. An independent statistician will randomise equal numbers within each block to the intervention and routine arms.

8.2 Outcome Measures

The primary endpoint will be aciclovir administration within 6 hours and an LP performed within 12 hours for patients with a suspected CNS infection.
The outcome measures will assess all primary and secondary outcomes as detailed above and a time to event analysis will be performed for each of these.

8.3 Sample Size and Analysis

Using pilot data from 315 patients across 26 hospitals in 4 deaneries we have estimated the standard deviations of the deanery and hospital random effects to be 0.244 and 1.108, respectively, and the current proportion of compliance under treatment as usual as 0.05. Using these estimates, the table below shows the power of the likelihood ratio test for a significant difference between standard care and the intervention as a function of m, the number of patients recruited per hospital, and p, the compliance proportion under the intervention (Table 2).

Table 2.

	p		
m	0.15	0.20	0.25
10	0.487	0.707	0.845
15	0.547	0.777	0.907
20	0.590	0.809	0.921
25	0.606	0.832	0.937

On the basis of these results, recruiting 20 patients per hospital should achieve a power of at least 0.8 when the compliance proportion under the intervention is 0.20. To allow for possible under-recruitment in some of the smaller hospitals, we will therefore seek to recruit 25 patients per hospital, but will close recruitment when we have achieved a total of at least 560 patients with at least 20 patients from each hospital.

We will analyse the results in STATA or R using a generalised linear mixed model (16) with binomial errors, logistic link, fixed effects for blocks and treatments, random effects for deanery and for hospital.

9 ETHICAL CONSIDERATIONS

9.1 Ethical Considerations

This study is encouraging 'best' healthcare in both the intervention and the control arms. The study will abide by the principles of the Research Governance Framework and ICH GCP. The study intervention will target clinicians and pseudo-anonymised patient data will be used to assess changes in clinical practice. Patients' records will be viewed by a member of NHS staff working at that site with medical information extracted using a CRF which will record a unique study number and patient date of birth but no other identifiers will be recorded.

9.2 Ethical Approval

The trial protocol has received the favourable opinion from Preston North West Multi-centre Research Ethics Committee (MREC) and will undergo site specific assessment (SSA) by completing section C of the REC application form.

10.3 Regulatory Approval

R&D approvals will be sought from each research sites before commencement of the study. Furthermore, an International Standard Randomised Controlled Trial Number (ISRCTN) number will be in place before the first site is actively opened.

10 TRIAL MONITORING

This study will be monitored via a central risk based approach as per the agreed monitoring plan.

10.1 Risk Assessment

An appropriate risk assessment will be completed and its resulting procedures followed during the study. As this study does not directly involve patients, or affect their care, this study will have low to zero risk attached to it.

10.2 Source Documents

Source data is all information, original records of clinical findings, observations, or other activities in a clinical trial necessary for the reconstruction and evaluation of the trial. Each participating site will maintain appropriate medical and research records for this trial, in compliance with ICH E6 GCP, Section 4.9 and regulatory and institutional requirements for the protection of confidentiality of subjects.

10.3 Data Capture Methods

Data collection

As we have done previously, a combination of methods to identify patients with suspected encephalitis involving screening of electronic sources (2, 3):

- Laboratory records for patients who have had a CSF sample taken;
- Pharmacy records for patients who have received intravenous aciclovir;
- Radiology records where 'suspected encephalitis' or other potential marker terms are used; and
- Hospital discharge codes.

All methods will be applied across all sites to minimize identification biases associated with any one method.

Trained research nurses will undertake retrospective case identification and data collection at each hospital site. The records of suspected and confirmed cases will be reviewed and data collected using a standardised form to document processes and selected outcomes of care. Data will be collected for the 12 months prior to and 12 months after the intervention period. It is unlikely that masked allocation to intervention or control hospital sites will be feasible during data collection but the feasibility of this will be reviewed.

The date and time from initial presentation to hospital, either via accident and emergency or medical admissions, will be taken as the starting point for data extraction and outcome measurement. As patients may be transferred between centres, patients within both groups will be characterised and the data analysed as per an intention to treat analysis.

Clinical data collection will be based upon the audit tool developed for the National Guideline. Apart from basic demographic data (for example date of birth and sex), no patient identifiers will be used, so that only sufficiently anonymised case records are transferred out of hospital settings for analysis. No one outside of the direct healthcare team will have access to any patient identifiable data as this will be pseudo-anonymised at site.

10.3.1 Case Report Forms

The study CRF is the primary data collection instrument for the study. All data requested on the CRF must be recorded. Data should be entered directly into OpenClinica ™ with paper CRFs to be used only in exceptional circumstances. All missing data must be explained with “N/D” for procedure not done, “UNK” for unknown and “N/A” for not applicable. If using a paper CRF, all entries should be printed legibly in black ink. If any entry error has been made, to correct such an error, draw a single straight line through the incorrect entry and enter the correct data above it. All such changes must be initialled and dated. Do not erase of white out errors. For clarification of illegible or uncertain entries, print the clarification above the item, then initial and date it.

10.4 Data checks

Data stored at Brain Infections UK coordinating centre will be checked for missing and unusual values (range checks) and checked for consistency within participants over time. Any suspect data will be returned to the site in the form of data queries. Data query forms will be produced at the Brain Infections UK coordinating centre from a password protected central web based database (OpenClinica ™) and sent either electronically or through the post to a named individual (as listed on the site delegation log). Sites will respond to the queries providing an explanation/resolution to the discrepancies and return the data query forms to Brain Infections UK coordinating centre. The forms will then be filed and the appropriate corrections made on the database. There are a number of monitoring features in place at the Brain Infections UK coordinating centre to ensure reliability and validity of study data.

10.4.1 Direct access to data

In order to perform their role effectively, monitors and persons involved in Quality Assurance and Inspection will need direct access to primary subject data, eg patient records, laboratory reports, appointment books, etc. As this affects the patient’s confidentiality, permission will be given by the principal investigator (PI) at the site.

10.4.2 Confidentiality

Individual participant medical information obtained as a result of this study is considered confidential and disclosure to third parties is prohibited. CRFs will be labelled with the patient’s initials and unique study screening and/or subject identity (ID) number before leaving site, however, data should be entered directly into OpenClinica™ and paper CRFs should be used as a last resort only. The Brain Infections UK coordinating centre will preserve the confidentiality of participants taking part in the study (only study number, sex and date of birth will be held) and The University of Liverpool is registered as a Data Controller with the Information Commissioners Office and this study is in full compliance of the Data Protection Act.

10.4.3 Quality Assurance and Quality Control of Data

This study has undergone a risk assessment, the outcome of which indicates this to be a low risk study. As such, site visits will be conducted and source data verification performed if indicated as a result of central monitoring processes. To this end:

- The Principal Investigator, and designated staff from each centre will attend the study initiation meeting, coordinated by Brain Infections UK coordinating centre in conjunction with co-lead investigators.
- The trial coordinator (or delegated person), is to verify appropriate approvals are in place prior to the initiation of a site, and that the relevant personnel have attended study specific training.
- The trial coordinator (or delegated person), is to monitor screening, recruitment and drop-out rates between centres.
- The trial coordinator (or delegated person), is to conduct data entry consistency check and follow up data queries.

Local quality control (QC) will include:

- Data will be evaluated for compliance with protocol and accuracy in relation to source documents
- The study will be conducted in accordance with procedures identified in the protocol.

10.5 Records Retention

The Investigator at each investigational site must make arrangements to store the essential trial documents, (as defined in Essential Documents for the Conduct of a Clinical Trial (ICH E6, Guideline for Good Clinical Practice)) including the Investigator Trial File, until the Brain Infections UK coordinating centre informs the Investigator that the documents are no longer to be retained, or for a maximum period of 15 years (whichever is soonest).

In addition, the Investigator is responsible for archiving of all relevant source documents so that the trial data can be compared against source data after completion of the trial (eg in case of inspection from authorities). The Investigator is required to ensure the continued storage of the documents, even if the Investigator, for example, leaves the clinic/practice or retires before the end of required storage period. Delegation must be documented in writing.

The Brain Infections UK coordinating centre undertakes to store any originally completed paper CRFs and separate copies of the above documents for the same period, except for source documents pertaining to the individual investigational site, which are kept by the investigator only. The Brain Infections UK coordinating centre will archive the documents in compliance with ICH GCP utilising the Records Management Service of the University of Liverpool. All electronic CRFs and study data will be archived onto an appropriate media for long term accessible storage. Hard copies of data will be boxed and transferred to specially renovated, secure, premises where unique reference numbers are applied to enable confidentiality, tracking and retrieval.

11 INDEMNITY

ENCEPH UK – Intervention RCT is co-sponsored by University of Liverpool and Walton Centre Foundation Trust and co-ordinated by the Brain Infections UK coordinating centre in the University of Liverpool. The University of Liverpool and The Walton Centre Foundation Trust does not hold insurance against claims for compensation for injury caused by participation in a clinical study and they cannot offer any indemnity. However, in terms of liability, NHS Trust and Non-Trust Hospitals have a duty of care to patients treated, whether or not the patient is taking part in a clinical study, and they are legally liable for the negligent acts and omission of their employees. Compensation is therefore available in the event of clinical negligence being proven.

Clinical negligence is defined as:
"A breach of duty of care by members of the health care professions employed by NHS bodies or by others consequent on decisions or judgments made by members of those professions acting in their professional capacity in the course of their employment, and which are admitted as negligent by the employer or are determined as such through the legal process".

12 FINANCIAL ARRANGEMENTS

This study is funded by National Institute for Health Research (NIHR) Programme Grant for Applied Research.

13 TRIAL COMMITTEES

With this study being part of a larger programme the below meeting structure has been devised to facilitate effective communication and support.

13.1 Programme Steering Group

The Programme Steering Group will consist of an independent chairperson, an expert in the field of medical neurology, a biostatistician with expertise in randomised cluster trials, an epidemiologist, co-applicants and key collaborators. The role is to provide overall supervision for the overall programme and provide advice through its independent Chairman. The ultimate decision for the continuation of the trial lies with the Programme Steering Group.

13.2 Programme Management Group

The Programme Management Group will bring together the leads of the study management group to provide a monthly update on progress and to provide support and advice to any issues that may occur.

13.3 Study Management Group

The individual study management group will be responsible for the day to day running of the trial and will meet on a fortnightly basis throughout the duration of the study.

14 PUBLICATION

The results from different centres will be analysed together and published as soon as possible. Individual clinicians must undertake not to submit any part of their individual data for publication without the prior consent of the Programme Management Group.

A detailed publication policy will be drawn up and agreed with members of the programme steering group and will be provided to sites as part of site set-up.

Regular updates regarding the study will be published on the ENCEPH-UK website (www.EncephUK.org). Where consent has been obtained, regular e-mails will be sent to participants to inform them when the website has been updated. These emails will also be sent to all participating sites.

15 PROTOCOL AMENDMENTS

List all amendments by number since the first final protocol was released. State where, how and why the protocol was changed. Include anything an MREC has had to approve.

15.1 Version 1 (dd/mon/yyyy)

Original Approved version.

16 REFERENCES

1. Jackson AC. Chapter 92 - VIRAL MENINGITIS AND ENCEPHALITIS. Neurology and Clinical Neuroscience. Philadelphia, Mosby**:** 2007**;**1249-1259.

2. Micheal BD, Sidhu M, Stoeter D, Roberts M, Beeching NJ, Bonington A, Hart IJ, Kneen R, Miller A, Solomon T. Acute central nervous system infections in adults — a retrospective cohort study in the NHS North West region. *Q J Med* 2010;103:749–758

3. Kelly C, Sohal A, Michael BD, Riordan A, Solomon T, Kneen R. Suboptimal management of central nervous system infections in children: a multi-centre retrospective study. *BMC Pediatrics* 2012:12:145-152

4. Bell DJ, Suckling R, Rothburn MM, Blanchard T, Stoeter D, Michael B, Cooke RPD, Kneen R, Solomon T. Management of Suspected Herpes Simplex Virus Encephalitis in Adults in a UK Teaching Hospital. *Clinical medicine* 2008; In Press.

5. Kneen R, Jakka S, Mithyantha R, Riordon A, Solomon T. The management of infants and children who receive aciclovir for suspected viral encephalitis. *Archives of Diseases in Childhood* 2008 (submitted); Corrections being made.

6. Minton J, Clayton J, Sandoe J, Mc Gann H, Wilcox M. Improving early management of bloodstream infection: a quality improvement project. *BMJ* 2008;336**:**440-443.

7. Solomon T, Hart IJ, Beeching NJ. Viral encephalitis: a clinician's guide. *Pract Neurol* 2007;7**:**288-305.

8. Kneen R, Solomon T, Appleton R. The role of lumbar puncture in children with suspected central nervous system infection. *BMC Pediatrics* 2002;2**:**8.

9. Heyderman RS, Lambert HP, O'Sullivan I, Stuart JM, Taylor BL, Wall RA. Early management of suspected bacterial meningitis and meningococcal septicaemia in adults. *J Infect* 2003;46**:**75-77.

10. Hasbun R, Abrahams J, Jekel J, Quagliarello VJ. Computed tomography of the head before lumbar puncture in adults with suspected meningitis. *New England Journal of Medicine* 2001;345**:**1727-1733.

11. Heyderman RS. Early management of suspected bacterial meningitis and meningococcal septicaemia in immunocompetent adults--second edition. *J Infect* 2005;50**:**373-374.

12. Solomon T, Michael BD, Smith PE, Sanderson F, Davies NWS, Hart, IJ, Holland M, Easton A, Buckley C, Kneen R, Beeching NJ, On behalf of the National Encephalitis Guidelines Development and Stakeholder Groups. Management of suspected viral encephalitis in adults - Association of British Neurologists and British Infection Association National Guidelines. *J Infect* 2012;64:347-373

13. Brazier J, Roberts J, Deverill M. The estimation of a preference-based measure of health from the SF-36. *J Health Econ* 2002;21**:**271-292.

14. Fenwick E, Claxton K, Sculpher M. Representing uncertainty: the role of cost effectiveness acceptability curves. *Health Econ* 2001;10**:**779-787.

15. Chaudhary MA, Stearns SC. Estimating confidence intervals for cost effectiveness ratios: an example from a randomized trial. *Stat Med* 1996;15**:**1447-1458.

16. Breslow NE and Clayton DG. Approximate inference in generalized linear mixed models. *Journal of the American Statistical Association* 1993;88:9-25
